# CT prediction of malignancy in part-solid pulmonary nodules based on vascular interruption and distortion

**DOI:** 10.3389/fmed.2026.1811922

**Published:** 2026-04-29

**Authors:** Silin Du, Feipeng Song, Ruiyu Lin, Fajin Lv

**Affiliations:** 1 Department of Radiology, The First Affiliated Hospital of Chongqing Medical University, Chongqing, China; 2 Department of Radiology, The Second Hospital of Shanxi Medical University, Shanxi, China

**Keywords:** computed tomography, lung adenocarcinoma, part-solid pulmonary nodule, prediction model, vascular distortion, vascular interruption

## Abstract

**Objective:**

To develop and validate computed tomography (CT)-based prediction models for malignancy in part-solid pulmonary nodules (PSNs).

**Methods:**

In this retrospective study, 204 surgically resected PSNs (107 malignant, 97 benign) were analyzed. Clinical data and CT morphological features were evaluated. Vascular patterns were classified into five types (I-V). Quantitative vascular counts (N1-N5, TN) were recorded. Nodules were randomly split into training (*n* = 143) and testing (*n* = 61) cohorts. Three logistic regression models were constructed: Model 1 (baseline clinical and morphological features), Model 2 (Model 1 + qualitative vascular types IV and V), and Model 3 (Model 1 + quantitative vessel counts N1-N3). Model performance was assessed using the area under the receiver operating characteristic curve (AUC), calibration (Hosmer-Lemeshow test), and clinical utility (decision curve analysis).

**Results:**

Malignant nodules were associated with older age (59 ± 10 vs. 56 ± 11 years, *p* = 0.018), female predominance (62.6% vs. 43.3%, *P* = 0.006), and specific CT features including irregular shape, lobulation, spiculation, vacuole sign, and pleural indentation (all *P* < 0.05). Vascular patterns IV (interruption) and V (distortion) were significantly more prevalent in malignant nodules (43.9% vs. 14.4%, and 51.4% vs. 5.2%, respectively; both *P* < 0.001). Quantitative counts of interrupted (N4) and distorted (N5) vessels were also significantly higher in malignancies (*P* < 0.001). In multivariable analysis, Model 2, incorporating vascular types IV and V, demonstrated superior predictive performance with a training AUC of 0.916 (95% CI: 0.872–0.960) and a testing AUC of 0.898 (95% CI: 0.821–0.974), significantly outperforming Model 1 (AUC 0.860/0.827) and Model 3 (AUC 0.866/0.823) (DeLong test, *P* = 0.012 and *P* = 0.040). Model 2 also showed excellent calibration and provided the highest net clinical benefit across a wide range of threshold probabilities.

**Conclusion:**

Qualitative CT assessment of vascular interruption and distortion (types IV and V) significantly improves the prediction of malignancy in PSNs over conventional morphological features alone. A model integrating these vascular patterns offers excellent diagnostic accuracy and clinical utility, potentially aiding in the preoperative risk stratification of PSNs.

## Highlights

- Pulmonary vessels are frequently observed within both benign and malignant part-solid pulmonary nodules.
- Qualitative CT assessment of vascular interruption and distortion (types IV and V) significantly improves the prediction of malignancy in PSNs over conventional morphological features alone.
- A model integrating vascular interruption and distortion offers excellent diagnostic accuracy and clinical utility, potentially aiding in the preoperative risk stratification of PSNs.

## Introduction

The widespread adoption of low-dose computed tomography (LDCT) for lung cancer screening has led to a significant increase in the detection of pulmonary nodules, particularly subsolid nodules which include pure ground-glass nodules (GGNs) and part-solid nodules (PSNs) (1). PSNs, characterized by both ground-glass and solid components, harbor a substantially higher risk of malignancy compared to pure GGNs, with reported malignancy rates ranging from 10 to 60% (2, 3). Accurate preoperative differentiation between malignant and benign PSNs is therefore critical for clinical management, balancing the risks of unnecessary invasive procedures against the dangers of delayed diagnosis of lung cancer.

Current guidelines, such as those from the Fleischner Society, primarily rely on nodule size, solid component size, and growth over time to guide management decisions in PSN (1, 4). While effective, this morphological approach has limitations. Benign processes like organizing pneumonia or focal fibrosis can manifest as persistent PSNs, mimicking adenocarcinoma (5). Consequently, there is a pressing need for more refined imaging biomarkers to improve diagnostic specificity and risk stratification.

The tumor microenvironment, particularly angiogenesis and vascular remodeling, plays a fundamental role in the growth and progression of lung adenocarcinoma, which is the most common malignancy presenting as a PSN (6). High-resolution CT can visualize the relationship between intratumoral vessels and the nodule architecture. Emerging evidence suggests that specific vascular alterations, such as vessel interruption, distortion, and convergence, are closely associated with invasive malignant behavior (7–10). Recent studies have begun to quantify and classify these vascular features. Qualitative classifications based on vessel course—passing-by, passing-through, compressed, interrupted, and distorted—have shown promise in differentiating invasive pulmonary adenocarcinomas from preinvasive or benign lesions (8, 11). Furthermore, quantitative assessments, such as counting the number of specific vessel types within a nodule, have been explored as objective biomarkers (9, 11, 12). However, the comparative diagnostic value of qualitative versus quantitative vascular analysis, and their incremental benefit over standard clinical and morphological features, remains inadequately investigated, especially in a cohort of surgically resected PSNs with definitive pathological confirmation.

Therefore, this retrospective study aims to systematically evaluate the role of CT-based vascular analysis in the preoperative diagnosis of PSNs. We evaluated both quantitative and qualitative vascular features in PSNs, focusing on vascular counts and types of vascular signs, with particular attention to vascular interruption and distortion. Specifically, we seek to develop and validate prediction models that integrate these vascular features with conventional CT morphological and clinical characteristics to improve the accuracy of malignancy prediction, thereby facilitating more precise and personalized management strategies for patients with PSNs.

## Materials and methods

### Study subjects

**FIGURE 1:**
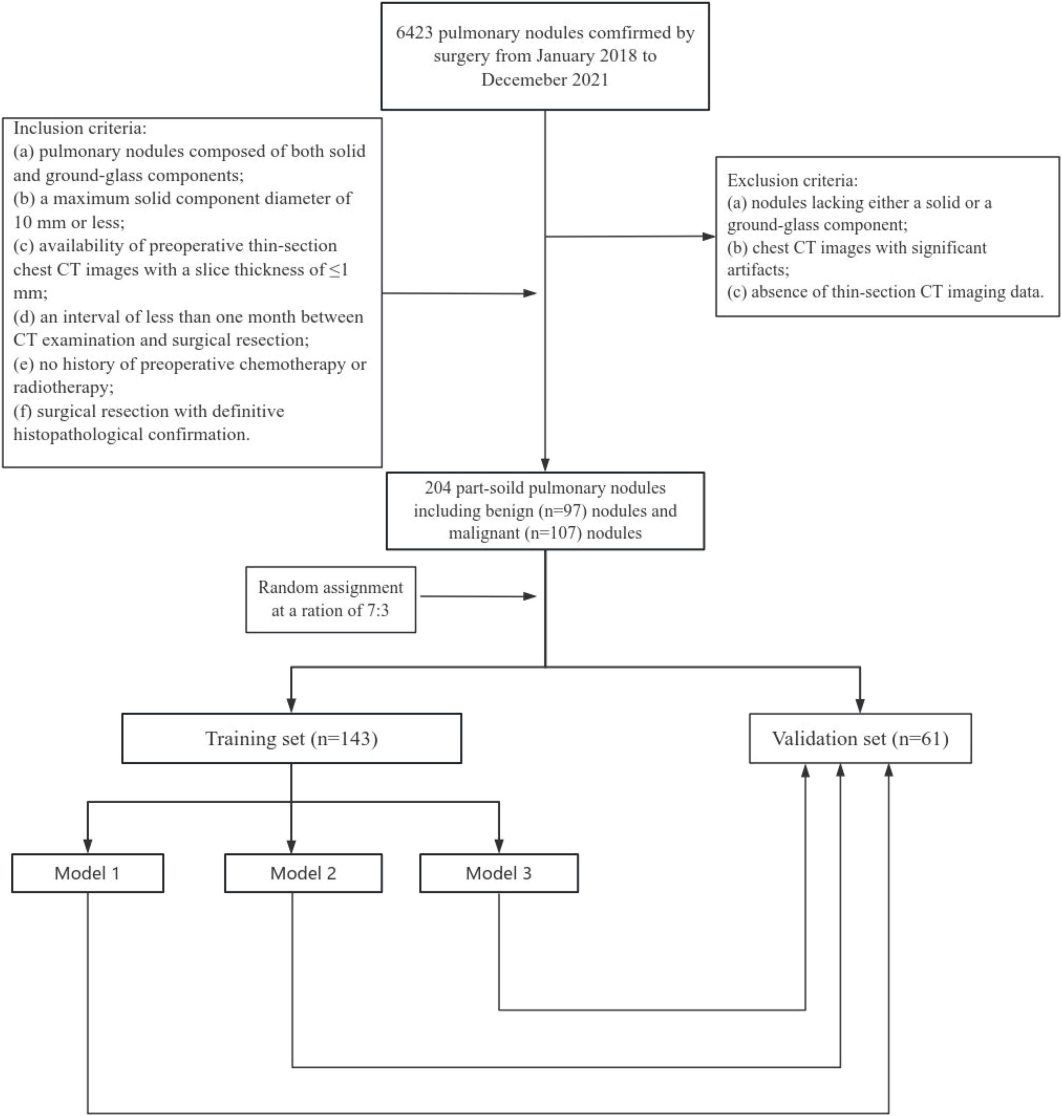
Flowchart of study population. This study included 204 part-solid pulmonary nodules including benign nodules and malignant nodules. Model 1 included clinical factors and standard morphologic CT features; Model 2 additionally included qualitative nodule-associated vascular features, and Model 3 incorporated the number of nodule-associated vessels based on Model 1.

This retrospective study was approved by the review board of our hospital and was exempted from the requirement of informed consent (2022-K346). All procedures involving participants were conducted in accordance with the Declaration of Helsinki (as revised in 2013). The study workflow is shown in Figure 1.

Between January 2018 and December 2021, 6,423 pulmonary nodules were surgically confirmed. Ultimately, a total of 194 patients with 204 part-solid pulmonary nodules were retrospectively enrolled. All nodules were classified as benign group or malignant group according to postoperative histopathological results. Preinvasive lesions were grouped as benign group based on pathological invasiveness, including adenocarcinoma in situ (AIS) and atypical adenomatous hyperplasia (AAH) (3, 13). The inclusion criteria were as follows: (a) pulmonary nodules composed of both solid and ground-glass components; (b) a maximum solid component diameter of 10 mm or less; (c) availability of preoperative thin-section chest CT images with a slice thickness of ≤ 1 mm; (d) an interval of less than 1 month between CT examination and surgical resection; (e) no history of preoperative chemotherapy or radiotherapy; (f) surgical resection with definitive histopathological confirmation. The exclusion criteria included: (a) nodules lacking either a solid or a ground-glass component; (b) chest CT images with significant artifacts; (c) absence of thin-section CT imaging data.

### CT Imaging parameters

All patients underwent unenhanced chest CT examinations using one of the following scanners: SOMATOM Definition Flash or SOMATOM Perspective (Siemens Healthineers, Erlangen, Germany), or Discovery CT750 HD (GE Healthcare, Milwaukee, WI, United States). Scans were performed with patients in the supine position during full inspiration, with both arms raised above the head. The scan range covered the region from the lung apex to the lung base. Although different CT acquisition protocols were employed, the main scanning parameters were generally consistent across examinations. These included a tube voltage of 110–130 kVp, tube current–time product of 50–140 mAs with automatic exposure control, detector collimation of 5 mm, pitch ranging from 1.0 to 1.1, gantry rotation time of 0.5 s, and a matrix size of 512 × 512. All images were reconstructed using a lung reconstruction kernel, with a slice thickness and reconstruction interval of either 0.625 mm or 1.0 mm.

### Clinical and CT evaluation of pulmonary nodules

All thin-section CT images were independently reviewed by two radiologists (S.D. with 11 years and R.L. and 5 years of experience in thoracic imaging). Image analysis was performed in a double-blinded manner. Any differences were resolved through consensus after discussion with a senior researcher (35 years of experience, F. L). Clinical variables, including age, sex, body mass index, smoking status, and personal or family history of malignancy, were obtained from the medical records. Serum carcinoembryonic antigen (CEA) levels were measured before surgery in all patients with identified pulmonary nodules.

CT images were reviewed on a dedicated post-processing workstation (United Imaging Healthcare, Shanghai, China) using mediastinal (width, 350–400 HU; level, 20–40 HU) and lung (width, 1,200–1,600 HU; level, -500 to -700 HU) window settings. All PSNs were evaluated on the lung window. Conventional CT features were systematically assessed. Nodule location (subpleural or non-subpleural) was recorded, with subpleural nodules defined as those with a minimum distance of less than 1 cm between the lesion margin and the visceral pleura. Nodule size was measured as the maximum diameter (MD) on axial lung window images. The maximum diameter of the solid component(MDs) was also recorded, and the consolidation-to-tumor ratio (CTR) was calculated accordingly. Nodule density was evaluated by separately assessing the ground-glass component (CTm) and the solid component (CTs). Morphologic features included nodule shape, categorized as regular (round or oval) or irregular, defined by an uneven or non-classifiable contour. Marginal characteristics, including spiculation and lobulation, were also noted. Internal features comprised the presence of vacuole sign, defined as round or oval air attenuation areas within the solid component, as well as abnormal air bronchograms. External features focused on pleural indentation, identified as linear or cord-like structures connecting the solid component of the nodule to the pleura on lung window images.

### Assessment of nodule-associated vascular features

**FIGURE 2:**
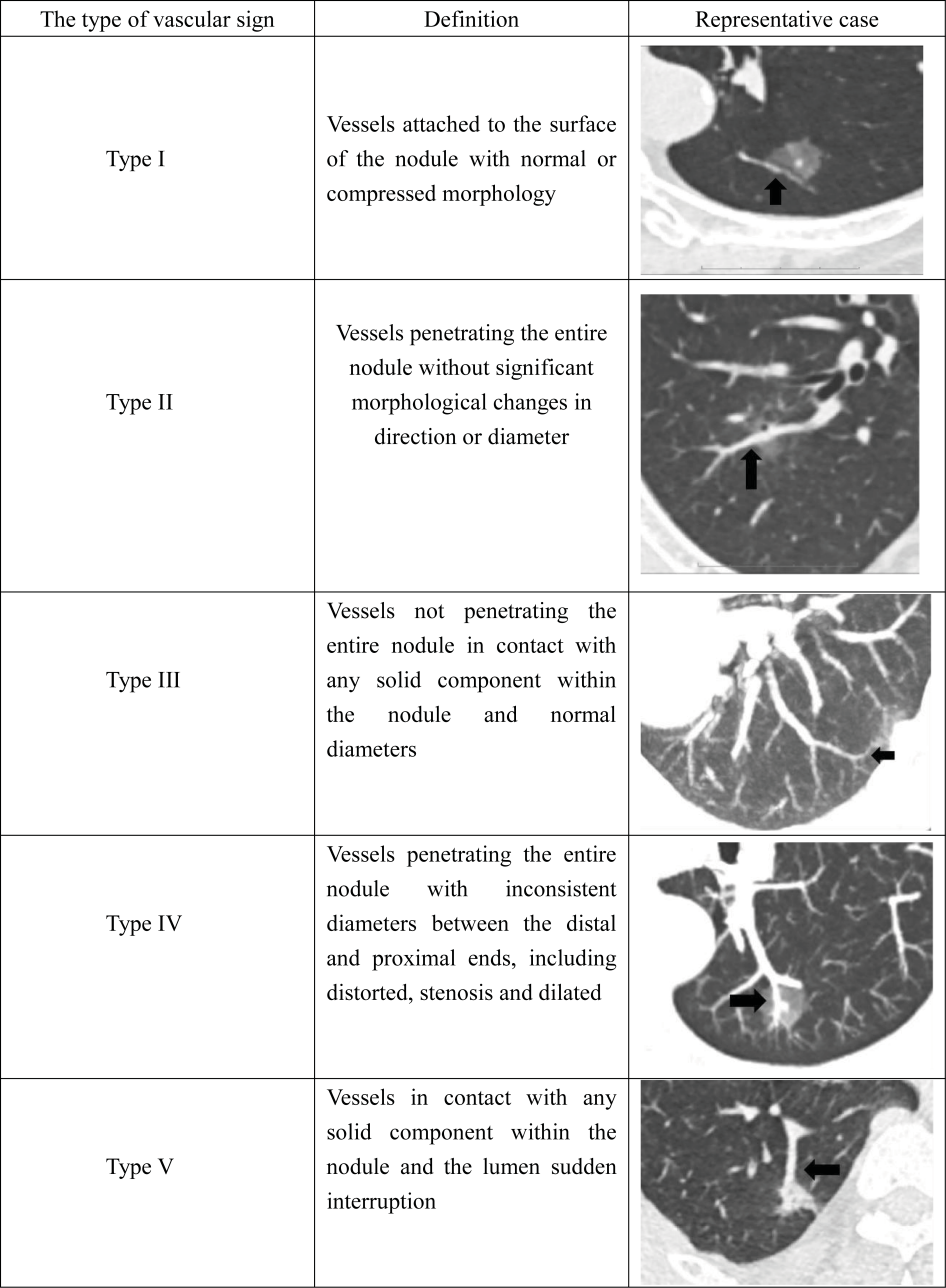
Classification and representative images of CT vascular sign.

Under normal physiological conditions, pulmonary vessels course from the hilum toward the lung periphery with a gradual decrease in caliber. Based on previously reported classification systems, nodule-associated vascular patterns associated with part-solid nodules were categorized into five types, as illustrated in Figure 2.

Type I (A1) was defined as vessels attached to the surface of the nodule without penetrating it.
Type II (A2) included vessels that traversed the entire nodule without apparent changes in direction or diameter, encompassing both entering vessels and their intranodular branches.
Type III (A3) referred to vessels that contacted any solid component within the nodule but did not pass through the entire lesion.
Type IV (A4) described vessels that penetrated the entire nodule while exhibiting caliber variation between proximal and distal segments, such as distortion, stenosis, or dilation, reflecting abnormal vascular morphology.
Type V (A5) included vessels in contact with a solid component that demonstrated interruption, defined as abrupt truncation or discontinuity of the vessel.

For each part-solid nodule, the number (N) of vessels corresponding to each vascular type was recorded. The total vascular number (TN) was calculated as the sum of all identified vascular types.

### Model development and performance evaluation

Data were randomly divided into training and testing cohorts at a 7:3 ratio. In the training group, the independent predictors of PSNs were selected through differential analysis. Variables with *p* < 0.05 in univariable logistic regression were then considered for multivariable logistic regression. A backward stepwise selection based on the Akashi Information Criterion (AIC), while excluding features with variance inflation factor (VIF) > 4.0. Multivariable logistic regression was used to establish the model. According to the regression coefficients of the corresponding features, three multivariable binary logistic regression models were constructed: Model 1 incorporated conventional morphological CT features; Model 2 additionally included categories of nodule-associated vascular patterns; and Model 3 incorporated the number of each type nodule-associated vessels based on Model 1.

The receiver operating characteristic (ROC) curve was used to evaluate the discriminative ability of the model, and the Delong test was used to assess the significance of the area under the curve (AUC). The calibration of the model was evaluated using a calibration curve and the Hosmer–Lemeshow goodness-of-fit test, and decision curve analysis (DCA) was used to assess the clinical utility of the model.

### Statistical analysis

All statistical analyses were performed using SPSS 26.0 and R software (version 4.2.2.^1^ Interobserver agreement was evaluated in a randomly selected subset of 30 cases from the overall cohort, including 15 benign and 15 malignant nodules. Agreement for categorical variables was assessed using Cohen’s kappa statistic, while consistency for continuous variables was evaluated using the intraclass correlation coefficient (ICC). The strength of agreement was interpreted according to established criteria: 0.41–0.60 as moderate, 0.61–0.80 as substantial, and 0.81–1.00 as almost perfect agreement. Normality of continuous variables was assessed using the Kolmogorov–Smirnov test. Normally distributed variables are presented as mean ± standard deviation and were compared using the Independent-Samples *t*-test, whereas non–normally distributed variables are presented as median (interquartile range) and were compared using the Mann–Whitney U test. Categorical variables are expressed as frequencies and percentages and were compared using the Chi-square test or Fisher’s exact test. A two-tailed *P* < 0.05 was considered statistically significant.

Logistic regression was performed using the software package “stats[R].” The “pROC[R]” software package was used to calculate the AUC and perform the DeLong test. The “ResourceSelection [R]” software package was used to perform the Hosmer–Lemeshow test, the “rmda[R]” package was used for decision curve analysis (DCA).

## Results

### Interobserver agreement

Interobserver agreement was assessed according to variable type. For continuous variables, including MD, MDs, CTm, CTs, and the counts of vascular sign types (NI–NV and TN), agreement was evaluated using the intraclass correlation coefficient (ICC). The ICC values between the two observers were as follows: MD (0.891), MDs (0.696), CTm (0.925), CTs (0.732), N1 (0.688), N2 (0.811), N3 (0.931), N4 (0.834), N5 (0.779), and TN (0.704). For categorical variables, including subpleural, boundary, shape, lobulation, spiculation, vacuole, bronchogram, pleural, and CT vascular sign types I–V, interobserver agreement was assessed using Cohen’s kappa statistic. The agreement was generally good to excellent, with kappa values as follows: subpleural (0.905), boundary (0.839), shape (1.000), lobulation (1.000), spiculation (0.865), vacuole (1.000), bronchogram (1.000), pleural (0.803), and for CT vascular sign types I–V: 1.000, 0.803, 0.783, 0.779, and 0.779, respectively. All values were statistically significant (all *P* < 0.001).

### Clinical characteristics

Baseline clinical characteristics of the 204 part-solid nodules, stratified by pathological diagnosis, are summarized in Table 1. Among the 204 nodules, 107 were pathologically confirmed as lung cancers, including 39 minimally invasive adenocarcinomas (MIA) and 68 invasive adenocarcinomas (IA). The 97 benign nodules consisted of 69 inflammatory pseudotumors, 25 Preinvasive lesions (14 AIS and 11 AAH), and 3 nodules with other histological diagnoses (1 lymphatic vessel leiomyoma and 2 tuberculosis).

**TABLE 1:** Clinical characteristics between benign and malignant group.

Characteristic	Groups		*P*-value
	Benign group *N* = 97	Malignant group *N* = 107	
Age, Mean ± SD	56 ± 11	59 ± 10	0.018*^1^
Gender, n (%)			0.006*^2^
Female	42 (43.3%)	67 (62.6%)	
Male	55 (56.7%)	40 (37.4%)	
BMI, Mean ± SD	23.3 ± 3.0	24.0 ± 2.8	0.074^1^
Smoking, n (%)			0.215^2^
Ever smoking	14 (14.4%)	8 (7.5%)	
Never smoking	68 (70.1%)	85 (79.4%)	
Smoking	15 (15.5%)	14 (13.1%)	
Cancer history, n (%)			0.449^3^
No	95 (97.9%)	102 (95.3%)	
Yes	2 (2.1%)	5 (4.7%)	
CEA, Mean ± SD	4.64 ± 20.61	2.21 ± 1.26	0.250^1^

^1^Welch Two Sample *t*-test.

^3^Fisher’s exact test.

^2^Pearson’s Chi-squared test.

*P < 0.05 indicates statistical significance.

The mean age was significantly older in the malignant part-solid nodule group than in the non-malignant group (59 ± 10 years vs. 56 ± 11 years, *P* = 0.018). A significant difference in sex distribution was also observed between the two groups (*P* = 0.006), with a higher proportion of female patients in the malignant group (62.6%) compared with the benign group (43.3%). No significant differences were identified between malignant and benign nodules with respect to body mass index(BMI) (24.0 ± 2.8 vs. 23.3 ± 3.0, *P* = 0.074), smoking status (*P* = 0.215), history of malignancy (*P* = 0.449), or mean serum carcinoembryonic antigen(CEA) levels (2.21 ± 1.26 vs. 4.64 ± 20.61, *P* = 0.250).

### Variables comparisons between benign and malignant groups

Conventional morphologic CT characteristics of the study population are summarized in Table 2. Among the 204 nodules, the distribution of subpleural location did not differ significantly between the malignant and benign groups (*P* = 0.754). Compared with benign nodules, malignant nodules more frequently demonstrated an irregular shape (66.4% vs. 28.9%, *P* < 0.001), lobulation (21.5% vs. 1.0%, *P* < 0.001), spiculation (23.4% vs. 8.2%, *P* = 0.003), vacuole sign (60.7% vs. 22.7%, *P* < 0.001), air bronchogram (16.8% vs. 5.2%, *P* = 0.009), and pleural indentation (41.1% vs. 27.8%, *P* = 0.047). A borderline difference was observed for boundary definition, which did not reach statistical significance (*P* = 0.095).

**TABLE 2:** Comparison the CT morphology feature between benign and malignant group.

Characteristic	Groups		*P*-value
	**Benign group *N* = 97**	**Malignant group *N* = 107**	
Subpleural, n (%)			0.754^1^
Absence	36 (37.1%)	42 (39.3%)	
Presence	61 (62.9%)	65 (60.7%)	
Shapes, n (%)			< 0.001*^1^
Regular	69 (71.1%)	36 (33.6%)	
Irregular	28 (28.9%)	71 (66.4%)	
Boundary, n (%)			0.095^1^
Fuzzy	24 (24.7%)	38 (35.5%)	
Clear	73 (75.3%)	69 (64.5%)	
Lobulation, n (%)			< 0.001*^1^
Absence	96 (99.0%)	84 (78.5%)	
Presence	1 (1.0%)	23 (21.5%)	
Spiculation, n (%)			0.003^1^
Absence	89 (91.8%)	82 (76.6%)	
Presence	8 (8.2%)	25 (23.4%)	
Vacuole sign, n (%)			< 0.001*^1^
Absence	75 (77.3%)	42 (39.3%)	
Presence	22 (22.7%)	65 (60.7%)	
Air-bronchogram, n (%)			0.009*^1^
Absence	92 (94.8%)	89 (83.2%)	
Presence	5 (5.2%)	18 (16.8%)	
Pleural indentation, n (%)			0.047*^1^
Absence	70 (72.2%)	63 (58.9%)	
Presence	27 (27.8%)	44 (41.1%)	
CTm(HU)	-622 ± 133	-580 ± 143	0.031*^2^
CTs(HU)	-101 ± 142	-136 ± 159	0.097^2^
MD(mm)	14.0 ± 4.7	14.5 ± 4.6	0.445^2^
MDS(mm)	5.62 ± 2.39	5.08 ± 2.04	0.090^2^
CTR(mm)	0.42 ± 0.15	0.37 ± 0.15	0.029*^2^

^1^Pearson’s Chi-squared test.

^2^Welch two sample *t*-test. CTm, the CT value of ground glass; CTs, the CT value of solid component; MD, maximum diameter of the whole nodule; MDs, maximum diameter of solid component; CTR, consolidation-to-tumor ratio.

*P < 0.05 indicates statistical significance.

For continuous variables, the mean CT attenuation of the ground-glass component was lower in benign nodules than in malignant nodules (-622 ± 133 HU vs. -580 ± 143 HU, *P* = 0.031). In contrast, the mean consolidation-to-tumor ratio(CTR) was higher in benign nodules compared with malignant nodules (0.42 ± 0.15 vs. 0.37 ± 0.15, *P* = 0.029). No significant differences were identified in CT attenuation of the solid component (*P* = 0.097), maximum diameter (*P* = 0.445), or maximum diameter of the solid component (*P* = 0.090).

The distribution of nodule-associated vascular patterns is summarized in Table 3. No statistically significant differences were observed between the benign and malignant groups for vascular types I (*P* = 0.097), II (*P* = 0.087), or III (*P* = 0.068). Notably, Vascular type III was present in both groups. In contrast, significant differences were identified for vascular types IV and V (both *P* < 0.001). The prevalence of type IV was 14.4% in the benign group and 43.9% in the malignant group. Similarly, type V was observed in 5.2% of benign nodules compared with 51.4% of malignant nodules.

**TABLE 3:** Comparison the types of CT vascular between benign and malignant group.

Characteristic	Groups		*P*-value
	Benign group *N* = 97	Malignant group *N* = 107	
A1, n (%)			0.097^1^
Absence	8 (8.2%)	17 (15.9%)	
Presence	89 (91.8%)	90 (84.1%)	
A2, n (%)			0.087^1^
Absence	64 (66.0%)	58 (54.2%)	
Presence	33 (34.0%)	49 (45.8%)	
A3, n (%)			0.068^1^
Absence	16 (16.5%)	29 (27.1%)	
Presence	81 (83.5%)	78 (72.9%)	
A4, n (%)			< 0.001*^1^
Absence	83 (85.6%)	60 (56.1%)	
Presence	14 (14.4%)	47 (43.9%)	
A5, n (%)			< 0.001*^1^
Absence	92 (94.8%)	52 (48.6%)	
Presence	5 (5.2%)	55 (51.4%)	

^1^Pearson’s Chi-squared test. *P* < 0.05 was considered statistically significant. A1, type I; A2, type II; A3, type III; A4, type IV; A5, type V.

*P < 0.05 indicates statistical significance.

**FIGURE 3:**
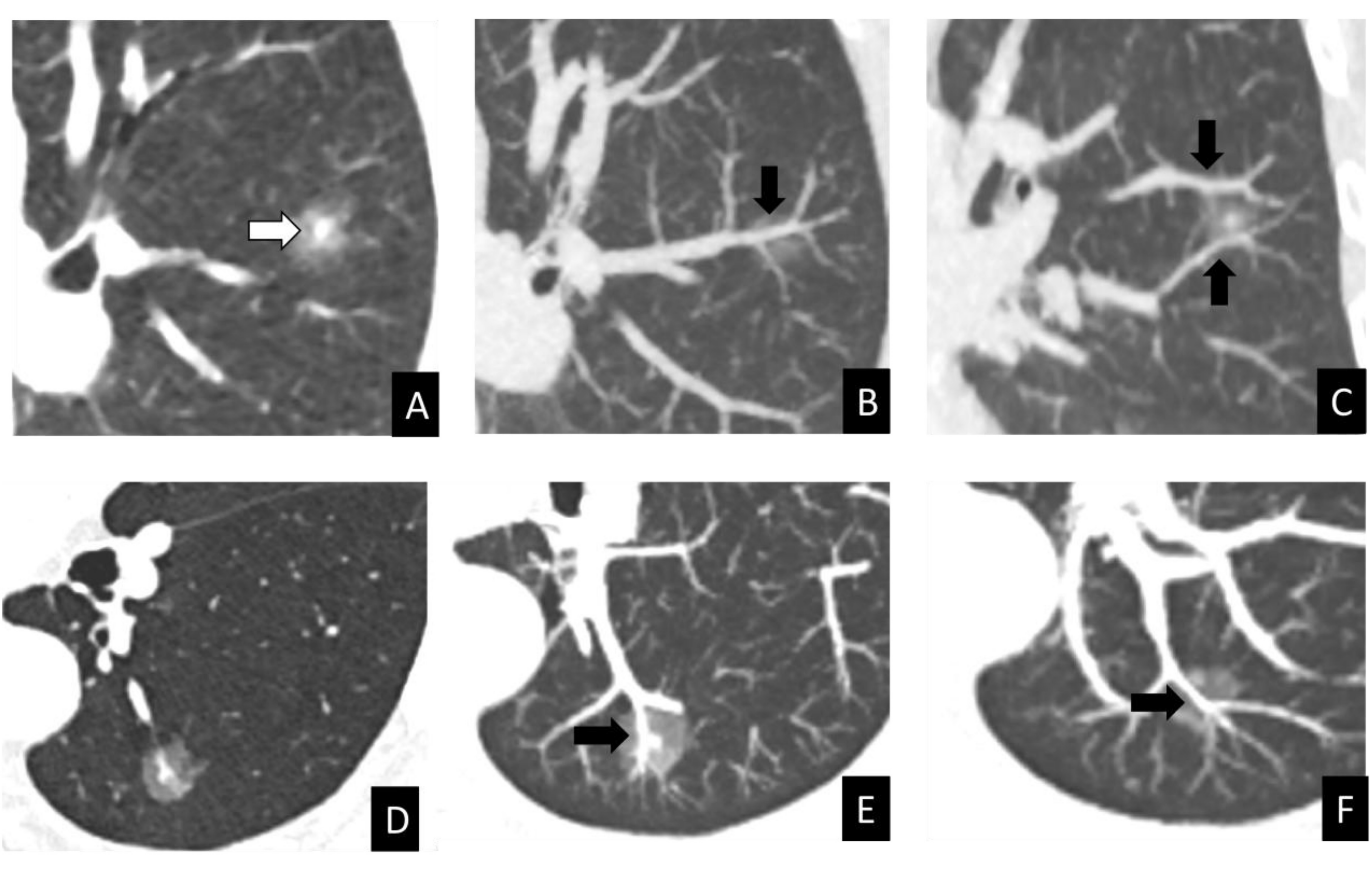
CT vascular signs in benign and malignant PSNs with similar morphological features. **(A–C)** Inflammatory nodule in a 68-year-old female. Axial CT images in lung window setting **(A)** show a PSN in the left upper lobe with irregular shape, lobulation. The MIP image **(B)** reveals a type II CT vascular sign (black arrow, N2 = 1). The MIP image **(C)** reveals a type I CT vascular sign (black arrow, N1 = 2). **(D–F)** Minimally invasive adenocarcinoma in a 64-year-old female. Axial CT images in lung window settings **(D)** show a PSN in the left lower lobe with irregular shape, lobulation. The MIP image **(E,F)** display type IV CT vascular signs (black arrow, N4 = 2). MIP, maximum-intensity projection; PSN, part-solid pulmonary nodules; N, number.

**FIGURE 4:**
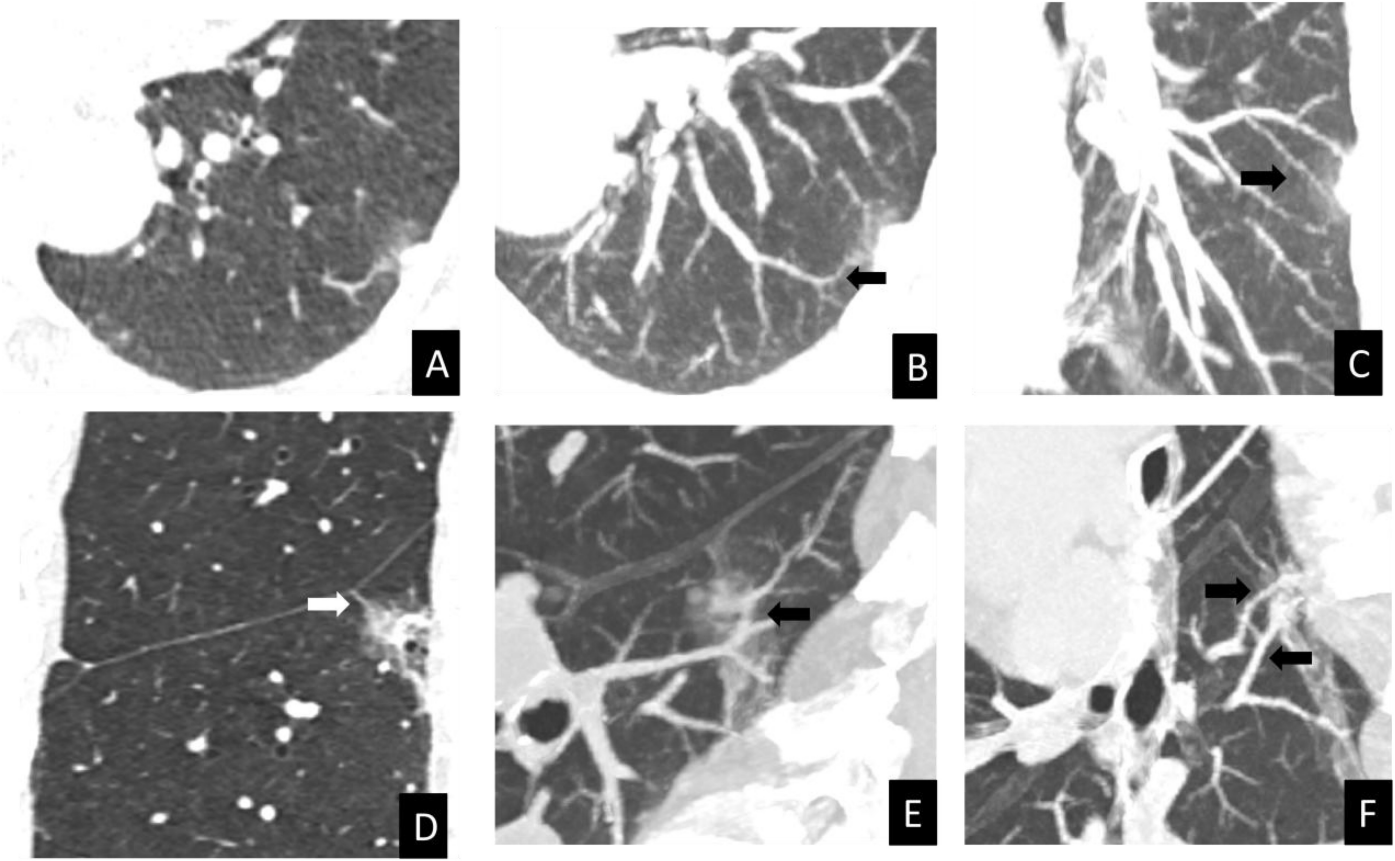
CT vascular signs in benign and malignant PSNs with similar morphological features. **(A–C)** Inflammatory nodule in a 67-year-old female. Axial CT images in lung window setting **(A)** shows a PSN in the left lower lobe with irregular shape, lobulation. The MIP image **(B)** reveals a type III CT vascular sign (black arrow, N3 = 1). The MIP image **(C)** reveals a type II CT vascular sign (black arrow, N2 = 1). **(D–F)** Invasive adenocarcinoma in a 76-year-old female. Coronal CT images in lung window setting **(D)** shows a PSN in the right lower lobe with irregular shape, lobulation and pleural traction (white arrow). The MIP image **(E)** displays type IV CT vascular signs (black arrow, N4 = 1). The MIP image **(F)** displays type V CT vascular signs (black arrow, N5 = 2). MIP, maximum-intensity projection; PSN, part-solid pulmonary nodules; N, number.

Quantitative comparisons of CT-assessed vascular counts are presented in Table 4. The mean numbers of nodule-associated vessels N1, N2, and N3 were comparable between the benign and malignant groups, with no statistically significant differences observed (*P* = 0.122, *P* = 0.082, and *P* = 0.585, respectively). In contrast, significant differences were identified for vascular N4 and N5, as well as for the total vascular number (TN). The malignant group demonstrated higher mean values for N4 (0.74 ± 1.05 vs. 0.22 ± 0.72, *P* < 0.001), N5 (0.64 ± 0.69 vs. 0.07 ± 0.36, *P* < 0.001), and TN (5.21 ± 1.47 vs. 4.23 ± 1.35, *P* < 0.001) compared with the benign group. These findings are illustrated in Figures 3, 4.

**TABLE 4:** Comparison the number of CT Vascular types between both groups.

Characteristic	Groups		*P*-value
	Benign group *N* = 97	Malignant group *N* = 107	
N1, Mean ± SD	1.79 ± 1.02	1.57 ± 1.04	0.122^1^
N2, Mean ± SD	0.55 ± 0.95	0.79 ± 1.08	0.082^1^
N3, Mean ± SD	1.57 ± 1.14	1.48 ± 1.22	0.585^1^
N4, Mean ± SD	0.22 ± 0.72	0.74 ± 1.05	< 0.001*^1^
N5, Mean ± SD	0.07 ± 0.36	0.64 ± 0.69	< 0.001*^1^
TN, Mean ± SD	4.23 ± 1.35	5.21 ± 1.47	< 0.001*^1^

^1^Welch Two Sample *t*-test. *P* < 0.05 was considered statistically significant. N1, the number of type I; N2, the number of type II; N3, the number of type III; N4, the number of type IV; N5, the number of type V; TN, the total number vessels of all types.

*P < 0.05 indicates statistical significance.

### Univariable and multivariable analysis for predicting malignancy

The nodules were randomly assigned to a training cohort (*n* = 143) and a testing cohort (*n* = 61) at a 7:3 ratio. Multivariable logistic regression analysis identified several variables independently associated with the outcome (Table 5). Higher CTm, MDS, CTR and CTs were associated with increased odds of malignancy, whereas higher N1, N2, and N3 values were inversely associated. Among irregular morphology, lobulation, vacuole sign, and vascular categories A4, A5 and TN were significantly associated with the malignancy, whereas spiculation and air bronchogram demonstrated borderline but non-significant associations.

**TABLE 5:** Univariable and multivariable logistic regression for predicting malignant.

Characteristic	Univariable		Multivariable	
	OR(95% CI)	*P*-value^1^	OR(95% CI)	*P*-value^1^
Age	0.97(0.94, 0.99)	0.019*	0.95(0.90, 1.00)	0.063
Gender				
Female	–	–		
Male	2.19(1.26, 3.87)	0.006**		
BMI	0.92(0.83, 1.01)	0.075		
Smoking				
Ever smoking	–	–		
Never smoking	0.46(0.17, 1.13)	0.097		
Smoking	0.61(0.19, 1.88)	0.396		
Cancer history				
No	–	–	1.28(1.00, 1.98)	0.247
Yes	0.43(0.06, 2.04)	0.319		
CEA	1.19(1.01, 1.47)	0.093		
Subpleural				
Absence	–	–		
Presence	1.09(0.62, 1.93)	0.754		
MD	0.98(0.92, 1.04)	0.443		
MDS	1.12(0.98, 1.27)	0.090	1.77(1.34, 2.48)	< 0.001***
CTR	1.00(1.00, 1.00)	0.101	1.01(1.00, 1.01)	0.012*
CTm	1.00(1.00, 1.00)	0.034*	1.00(0.99, 1.00)	0.010**
CTs	1.00(1.00, 1.00)	0.101	1.01(1.00, 1.01)	0.012*
Shapes				
Regular	–	– < 0.001***	0.27(0.09, 0.76)	0.015*
Irregular	0.21(0.11, 0.37)			
Boundary				
Fuzzy	–	–		
Clear	1.68(0.92, 3.10)	0.096		
Lobulation				
Absence	–	–	0.03(0.00, 0.28)	0.006**
Presence	0.04(0.00, 0.19)	0.002**		
Spiculation				
Absence	–	–		
Presence	0.29(0.12, 0.66)	0.005**	0.20(0.03, 1.12)	0.083
Vacuole sign				
Absence	–	–	0.16(0.05, 0.48)	0.002**
Presence	0.19(0.10, 0.35)	< 0.001***		
Air bronchogram				
Absence	–	–	0.16(0.02, 1.09)	0.083
Presence	0.27(0.09, 0.71)	0.013*		
Pleural indentation				
Absence	–	–		
Presence	0.55(0.30, 0.99)	0.048*		
A1				
Absence				
Presence	2.10(0.89, 5.38)	0.102		
A2				
Absence				
Presence	0.61(0.34, 1.07)	0.088		
A3				
Absence				
Presence	1.88(0.96, 3.80)	0.070		
A4				
Absence				
Presence	0.22(0.11, 0.42)	< 0.001***	0.25(0.07, 0.83)	0.027*
A5				
Absence				
Presence	0.05(0.02, 0.13)	< 0.001***	0.01(0.00, 0.04)	< 0.001***
N1	1.24(0.95, 1.63)	0.123	0.53(0.30, 0.92)	0.029*
N2	0.78(0.58, 1.03)	0.088	0.41(0.23, 0.67)	< 0.001***
N3	1.07(0.84, 1.35)	0.584	0.44(0.25, 0.70)	0.001**
N4	0.43(0.26, 0.65)	< 0.001***		
N5	0.10(0.04, 0.21)	< 0.001***		
TN	0.61(0.49, 0.75)	< 0.001***		

^1^*P < 0.05; **P < 0.01; ***P < 0.001. CI, Confidence Interval, OR, Odds Ratio. Only variables with *P* < 0.05 in the univariable analysis were entered into the multivariable logistic regression model.

### Development and performance evaluation of the prediction models

**FIGURE 5:**
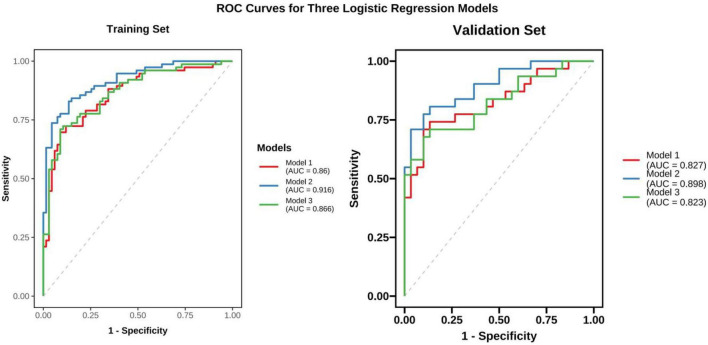
Receiver operating characteristic curves of classification models for predicting malignancy among part-solid nodules in the training and testing cohorts. Model 1, based on nodule conventional CT features; Model 2 nodule conventional CT features combined with nodule vascular sign; Model 3, nodule conventional CT features combined with the number of the vascular sign.

Three binary logistic regression models were developed to identify independent predictors of malignancy. After backward stepwise regression and multivariable logistic regression, it was found that CTm, MDS, CTR, CTs, irregular morphology, lobulation and vacuole sign (all VIFs were less than 2.0) were independent risk factors for predicting the malignant of PSNs in the construction of the model 1. The baseline logistic regression model, Model 1 a training set AUC of 0.860 (95% CI: 0.800–0.921) and a testing set AUC of 0.827 (95% CI: 0.722–0.932), with a sensitivity of 69.7% and specificity of 91.0% in both datasets, demonstrated good classification performance. Model 2, which added A4 and A5, achieved improved discrimination a training set AUC of 0.916 (95% CI: 0.872–0.960) and a testing set AUC of 0.898 (95% CI: 0.821–0.974), with a sensitivity of 82.9% and specificity of 86.6% in both datasets. Model 3, which included N1, N2, and N3, yielded a training set AUC of 0.866 (95% CI: 0.807–0.924) and a testing set AUC of 0.823 (95% CI: 0.717–0.929), with a sensitivity of 71.1% and specificity of 91.0% in both datasets (Figure 5). The DeLong test showed that Model 2 had a significantly highest outperformed than Model 1 (*P* = 0.012) and Model 3 (*P* = 0.040).

### Calibration of the models

**FIGURE 6:**
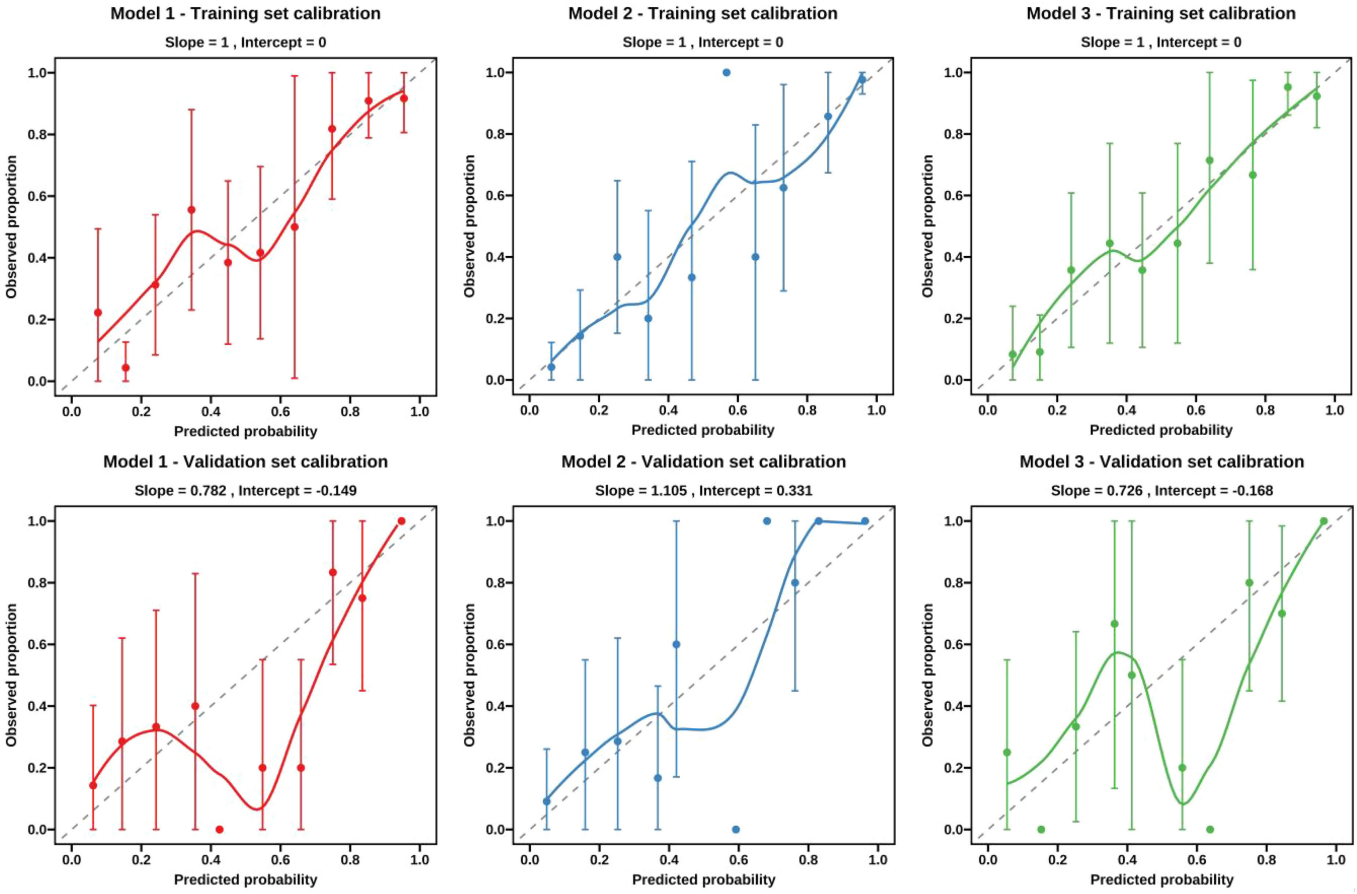
Calibration curves of the three models for malignancy prediction in part-solid nodules in the training and testing cohorts. Model 1, based on nodule conventional CT features; Model 2 nodule conventional CT features combined with nodule vascular sign; Model 3, nodule conventional CT features combined with the number of the vascular.

Figure 6 shows the calibration curves of the Model 1, Model 2, and Model 3 in the training set and testing set. The Hosmer–Lemeshow test indicated that the *P*-values for the Model 1, Model 2, and Model 3 in the training set and testing set were 0.603, 0.887, 0.802, and 0.178, 0.858, 0.159, respectively. These results demonstrated Model 2 achieved the good calibration and predictive accuracy of the constructed.

### Clinical utility of the models

**FIGURE 7:**
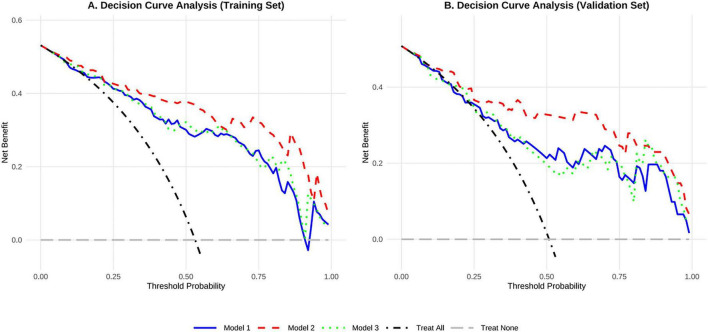
Decision curve analysis of the three models for malignancy prediction in part-solid nodules in the training and testing cohorts. Model 1, based on nodule conventional CT features; Model 2 nodule conventional CT features combined with nodule vascular sign; Model 3, nodule conventional CT features combined with the number of the vascular.

Figure 7 illustrates the decision curve analysis of the Model 1, Model 2, and Model 3 in the training set and testing set. Decisions based on these Model demonstrated an improvement in clinical net benefit, with the Model 2 providing a higher clinical net benefit for patients under most threshold probabilities when predicting PSNs malignancy.

## Discussion

This retrospective study investigated the utility of computed tomography (CT)-assessed vascular interruption and distortion, alongside conventional morphologic features, for predicting malignancy in PSNs. Our findings indicate that specific abnormal vascular patterns, particularly types IV and V representing vascular interruption and distortion, are strongly associated with invasive adenocarcinoma and serve as powerful independent predictors of malignancy. The integration of these vascular features into a predictive model significantly enhanced diagnostic performance compared to models based solely on conventional CT characteristics or quantitative vessel counts, with qualitative vascular patterns showing greater predictive value than vessel counts.

The baseline clinical and conventional CT characteristics of malignant and benign PSNs in our cohort align with established literature. Malignant nodules were associated with older age and female sex, consistent with known epidemiological trends for lung adenocarcinoma (14–16). Conventional CT features such as irregular shape, lobulated, spicule, and vacuole sign were significantly more frequent in malignant nodules, corroborating their well-documented role as indicators of invasiveness and malignancy (8, 17–24). The higher mean attenuation of the ground-glass component and solid component in malignant nodules further supports the concept that increasing density within a PSN often reflects progressive alveolar collapse and fibroblastic proliferation, hallmarks of invasive growth (3, 25). Based on these morphological predictors, Model 1 showed moderate performance (AUC = 0.827), prompting further investigation into the relationship between PSNs and surrounding vessels.

The most significant contribution of this study is the identification and validation of qualitative vascular patterns IV (interruption) and V (distortion) as powerful independent predictors. The rationale for their strong association with malignancy is rooted in tumor biology and angiogenesis. Vascular Distortion (Type IV): This pattern is primarily driven by tumor-associated angiogenesis and mechanical deformation. Malignant tumors secrete pro-angiogenic factors (e.g., VEGF), stimulating the formation of new, often tortuous and chaotic vessels (26). Simultaneously, the expanding tumor mass and associated fibrotic stroma mechanically push, pull, and distort the adjacent normal pulmonary vasculature (27, 28). The convergence and rigidiy of vessels toward the nodule, as seen in distortion, are classic signs of a desmoplastic stromal response (6). Vascular Interruption (Type V): This likely represents the consumption or destruction of pre-existing vessels by aggressively proliferating tumor cells (29). As a malignancy grows invasively, it can obstruct, encase, or directly invade pulmonary capillaries and small vessels, leading to their truncation on imaging. This is a radiological correlate of the tumor’s destructive local behavior. These findings align with reports by Gao et al. (11), who observed that vascular convergence signs were independent predictors of invasiveness in pure ground-glass nodules. They are also consistent with the work of Peng et al. (8), which described intratumoral vessel dilation, stenosis, and obstruction in solid nodules.

Unlike previous studies focusing mainly on vessel quantity, we also evaluated qualitative vascular patterns, particularly interruption and distortion. By integrating qualitative vascular patterns with conventional CT and clinical features into a predictive model, our study provides a more comprehensive and biologically meaningful approach to malignancy prediction in PSNs, which was not addressed in prior studies. In addition, the quantitative counts of interrupted (N4) and distorted (N5) vessels were also higher in malignancies, but the qualitative classification (A4/A5) when incorporated into predictive models proved more powerful. It may indicate that vessel quantity alone is a less specific marker, since benign inflammatory processes can also exhibit hypervascularity. Moreover, this suggests that the presence of aberrant vascular patterns is a more critical and biologically meaningful dichotomous indicator than a simple vessel count. This could be due to the limited prevalence of high-grade vascular patterns in our cohort, which consisted predominantly of early-stage malignant lesions, resulting in insufficient variability and reduced incremental predictive value. Alternatively, vessel quantity may not linearly correlate with malignant potential beyond a certain threshold, and it was not retained as an independent predictor in multivariate regression, as reported by Fu et al. (30) in ground-glass nodules. However, this findings was inconsistent with previous results that the number of intra-nodular vessels was related to the malignant degree of ground-glass nodules (11, 31) and may play an important role during the process of nodule growth (32). Thus, the role of vascular quantity differentiating between benign and malignant part-solid nodules is worthy of further study.

From a clinical standpoint, incorporating qualitative assessment of nodule-associated vascular morphology into routine CT interpretation may enhance risk stratification of part-solid pulmonary nodules. In daily practice, when the solid component is in direct contact with a vessel, malignancy should be regarded as a potential diagnosis. Such qualitative vascular information can complement conventional morphologic features and may assist clinicians in distinguishing benign or preinvasive nodules from lesions with higher malignant potential. Importantly, as these vascular features can be assessed on standard thin-section CT images without specialized software, they may be readily integrated into routine radiologic reporting of part-solid pulmonary nodules.

This study has several limitations. First, this was a retrospective single-center study lacking external validation. The findings need to be further validated in multicenter prospective studies. Second, all PSN cases included in this study were confirmed by surgery, which were clinically suspected as malignant characteristics of nodules being more prominent from the high-resolution CT imaging findings, resulting in a certain degree of selection bias in the sample. In practice, many disappeared benign PSNs were not included due to the lack of histological confirmation, resulting in a certain degree of selection bias in the sample. In the future, it may be necessary to include unproven PSNs, in order to make the model more representative of real-world conditions and more applicable to the large number of unproven long-term follow-up patients. Thirdly, this study was not combined with techniques such as machine learning and deep learning. The interpretation of CT features remains dependent on subjective visual assessment rather than artificial intelligence, the aim was to find a fast and convenient indicator for differentiating benign and malignant conditions without relying on any software. In future studies, a standardized scoring atlas with representative examples of vascular patterns may help improve consistency, and could be further supported by AI-assisted tools. Furthermore, in this study, the number of benign PSNs was small, and the proportion of the benign PSN was comparable to lung cancer only after being classified as non-neoplastic PSNs along with AAH and AIS. Therefore, we classify AAH and AIS into the benign group and will continue to expand the number of benign cases in the future.

## Conclusion

In conclusion, CT-based identification of vascular interruption and distortion (types IV and V) in PSNs is a strong and independent predictor of malignancy. Integrating these specific vascular features with conventional CT characteristics significantly improves the predictive accuracy for discriminating invasive adenocarcinoma from benign lesions. This approach offers a readily applicable, non-invasive method to enhance risk stratification in the management of PSNs, supporting more personalized clinical decisions.

## Data Availability

The original contributions presented in the study are included in the article/supplementary material, further inquiries can be directed to the corresponding author.

## Footnotes

1
https://www.r-project.org
